# Light–Root Microbiome Interactions in Vegetable Crops: From Photoreceptor Signaling to Exudate-Mediated Recruitment

**DOI:** 10.3390/ijms27167408

**Published:** 2026-08-19

**Authors:** Lidiia Samarina, Arysgul Turbekova, Serik Jantassov, Halil Demir, Farida Kozhakhmetova, Almagul Begalina, Renata Akzhunis, Khaiyrnisa Aisakulova

**Affiliations:** 1 Saken Seifullin Kazakh Agrotechnical University, 010000 Astana, Kazakhstan; q11111w2006@yandex.ru (L.S.); farida-_98@mail.ru (F.K.); alma.begalina@mail.ru (A.B.); renata.akzhunusova@mail.ru (R.A.); hairinissa@mail.ru (K.A.); 2 Kazakh Research Institute of Fruit and Vegetable Growing LLP, 050060 Almaty, Kazakhstan; 3 Department of Horticulture, Faculty of Agriculture, University of Akdeniz, 07058 Antalya, Turkey; hdemir@akdeniz.edu.tr

**Keywords:** protected cultivation, vegetable crops, rhizosphere, root microbiome, light intensity, red/far-red ratio, root exudates, LED lighting, disease suppression, synthetic microbial communities

## Abstract

In protected cultivation, light intensity, spectral quality, red/far-red ratio, photoperiod and diel fluctuation can alter the belowground biological environment by modifying carbon allocation, root architecture, root exudation, nutrient acquisition, immune tone and rhizosphere physicochemistry. Direct community-level evidence in vegetables remains sparse, but targeted experiments on bacterial colonization, arbuscular mycorrhizal symbiosis, beneficial fungi and root pathogens show that light can condition specific plant–microbe interactions. This review develops a molecular framework for light–root–microbiome interactions in protected vegetable crops and distinguishes direct community evidence, targeted colonization or symbiosis evidence, crop-specific indirect evidence and mechanistic analogues. We synthesize how photoreceptors and PIF-, HY5-, hormone- and immunity-related pathways regulate root niche construction, while also considering direct microbial photoreception. Experimental examples include tomato rhizosphere responses to shading, R:FR-dependent colonization by *Serratia plymuthica*, phyB–HY5–strigolactone control of tomato mycorrhization, light-intensity effects on lettuce–AMF interactions, spectrum-dependent *Trichoderma harzianum* colonization and light sensing by *Ralstonia pseudosolanacearum*. The evidence supports the view that light acts as a conditional regulator whose effects depend on crop genotype, microbial partner, substrate, nutrient status and developmental stage. Progress will require factorial lighting experiments coupled with exudomics, stable-isotope tracing, absolute microbial quantification, isolate genomics, synthetic communities and pathogen-challenge assays.

## 1. Introduction

In modern protected environments, growers can manipulate irradiance, photoperiod, spectral composition and canopy light distribution with increasing precision. Yet the biological consequences of light management extend belowground. Roots receive carbon, hormonal signals and developmental instructions from illuminated shoots, and root-associated microbes respond to the resulting changes in rhizodeposition, oxygen availability, pH, redox conditions and defense chemistry [1,2,3,4,5]. Particularly, tomato, cucumber, pepper, eggplant, lettuce, leafy greens, strawberry and other high-value vegetables are frequently grown under protected conditions with supplemental LEDs, shade nets, greenhouse coverings, high-density canopies or vertical-farm photoperiods. Soilborne pathogens, nutrient imbalances, salinity, biofilm dynamics in hydroponic channels, substrate reuse and variable inoculant establishment all influence productivity. The same management decision that changes canopy photosynthesis can plausibly change microbial recruitment in the rhizosphere and root endosphere [2,4,5].

The literature does not support a universal statement that a particular spectrum or light intensity improves the vegetable root microbiome. Community-level studies show that shading or light intensity can alter tomato rhizosphere bacterial and fungal composition [1,6]. Targeted experiments demonstrate that photoperiod and spectral quality can condition the establishment or function of defined bacterial and fungal partners, including PGPR, AMF and *Trichoderma*, while light can also be sensed directly by a root-infecting bacterial pathogen [2,7,8,9,10,11,12,13,14]. These targeted studies establish plausible mechanisms but should not be interpreted as proof that the entire microbiome shifts in the same direction. Evidence from rice, Arabidopsis, *Panax notoginseng* and other non-vegetable systems provides additional causal support for circadian regulation, microbiota feedback under low light and metabolite-mediated disease suppression [15,16,17,18,19,20,21,22].

At the molecular level, this field should be framed as a problem of shoot–root signal integration. Red, far-red, blue and UV-B cues are translated by photoreceptors into transcriptional and hormonal outputs, including HY5- and PIF-related modules, auxin redistribution, jasmonate–salicylate balance and defense-gating pathways. Thus, light-regulated microbiome assembly should be interpreted as the outcome of three interacting molecular filters: exudate chemistry, immune permissiveness and microbial response traits such as chemotaxis, biofilm formation and immune modulation [23,24,25,26,27,28,29]. To summarize the current knowledge in the field, this review focuses on root-associated microbiomes of vegetable crops in protected or intensively managed production systems.

## 2. Materials and Methods

### 2.1. Review Design and Scope

This review was designed as a structured critical review rather than a formal meta-analysis. The aim was to synthesize mechanistic and experimental evidence linking the aboveground light environment with root-associated microbiome assembly in protected and intensively managed vegetable production systems. The review focused on vegetable crops grown under conditions where light can be actively modified, including greenhouse cultivation, shade-net systems, vertical farming, hydroponics, soilless substrates, and controlled-environment agriculture. Studies on cereals, model plants, medicinal plants, and non-vegetable crops were not used as primary evidence for vegetable production, but were included selectively when they provided mechanistic insight into light-regulated rhizosphere processes that remain insufficiently studied in vegetables.

The root-associated microbiome was defined broadly to include rhizosphere soil or substrate, rhizoplane, root endosphere, and root-zone solution microbiota in hydroponic systems.

### 2.2. Literature Search Strategy

A structured literature search was conducted using Web of Science Core Collection, Scopus, PubMed, Google Scholar, and publisher databases including ScienceDirect, SpringerLink, Wiley Online Library, Oxford Academic, MDPI, Frontiers, and Nature Portfolio. The search was last updated on 24 July 2026.

“light” OR “light intensity” OR “shading” OR “shade” OR “photoperiod” OR “daily light integral” OR “LED” OR “light quality” OR “red far-red” OR “red/far-red ratio” OR “blue light” AND “root microbiome” OR “rhizosphere microbiome” OR “root endosphere” OR “rhizoplane” OR “root-associated microbiota” OR “bacterial community” OR “fungal community” OR “plant growth-promoting rhizobacteria” OR “biocontrol” AND “vegetable” OR “tomato” OR “cucumber” OR “lettuce” OR “pepper” OR “eggplant” OR “strawberry” OR “leafy greens” OR “greenhouse” OR “protected cultivation” OR “hydroponics” OR “controlled environment agriculture”.

Additional searches were performed for mechanistic terms, including “root exudates”, “chemotaxis”, “biofilm formation”, “carbon allocation”, “photosynthate allocation”, “flavonoids”, “organic acids”, “plant immunity”, “jasmonic acid”, “salicylic acid”, “phytochrome”, “HY5”, “PIF”, “circadian rhythm”, “disease suppressive microbiome”, “synthetic microbial community”, “mycorrhiza”, “arbuscular mycorrhizal fungi”, “vesicular-arbuscular mycorrhiza”, “*Glomus*”, “*Rhizophagus*”, “irradiance”, “photon irradiance”, “daylength”, “root colonization”, “microbial photoreceptor” and “LOV photoreceptor”. A supplementary targeted search combined the light-related terms above with “*Trichoderma*”, “beneficial fungal endophyte”, “mycoparasitic fungus”, “fungal photoreceptor”, “AMF colonization” and “multikingdom community”.

### 2.3. Inclusion and Exclusion Criteria

Studies were included when they met at least one of the following criteria: (i) they experimentally tested the effect of light intensity, light quality, photoperiod, shading, or diel light regime on root-associated microbial communities; (ii) they investigated vegetable root or rhizosphere microbiomes in protected or intensively managed cultivation systems; (iii) they provided mechanistic evidence linking light responses with root exudation, microbial recruitment, chemotaxis, biofilm formation, plant immunity, nutrient acquisition, or disease suppression; or (iv) they introduced methodological approaches relevant to future studies of light–root–microbiome interactions.

Primary emphasis was placed on experimental studies using 16S rRNA gene sequencing, ITS sequencing, shotgun metagenomics, culture-based microbial isolation, synthetic communities, exudate profiling, plant phenotyping, pathogen challenge assays, or nutrient use assays. Review articles were used to contextualize broader concepts, but mechanistic conclusions were based primarily on original experimental studies.

Studies were excluded if they focused only on aboveground phyllosphere microbiomes without root-zone relevance, if they examined light effects on plant physiology without any connection to roots, exudates, or microbiomes, or if they reported microbial community data without sufficient information on crop system, compartment, or experimental conditions.

### 2.4. Evidence Classification and Synthesis

The selected literature was organized into four evidence classes. First, direct community-level evidence comprised studies that manipulated light and profiled bacterial, fungal or multikingdom root-associated communities in a vegetable crop. Second, direct targeted evidence comprised studies that manipulated light and quantified colonization, establishment, behavior or function of a defined bacterium, beneficial fungus, AMF symbiosis or root pathogen in a vegetable crop. Third, crop-specific indirect evidence included vegetable studies on root exudates, microbiomes, disease suppression or hydroponic communities without light manipulation. Fourth, mechanistic analogues from model or non-vegetable systems were included when they provided causal insight into pathways plausibly relevant to vegetables. Fungal evidence was additionally evaluated by functional guild—AMF, non-mycorrhizal beneficial endophytes or mycoparasites, and pathogenic fungi—because colonization, nutrient exchange, antagonism and disease outcomes are not interchangeable endpoints.

Studies were considered particularly informative when they included metabolite complementation, microbial chemotaxis or biofilm assays, live versus sterilized substrate comparisons, pathogen-challenge assays, microbial isolation, synthetic-community testing, or absolute microbial quantification. Taxonomic shifts were interpreted cautiously, especially when based only on relative abundance data. Genera such as *Pseudomonas*, *Bacillus*, *Serratia*, *Enterobacter*, *Sphingomonas*, *Mortierella*, and *Trichoderma* were treated as functionally heterogeneous groups unless strain-level evidence was available.

### 2.5. Limitations of the Review Methodology

This review has several limitations. First, direct community-level evidence in vegetable crops remains limited, although the targeted literature on bacterial colonization, AMF symbiosis, beneficial fungi and root pathogens is broader than community sequencing studies alone suggest. Second, targeted single-strain or single-symbiont experiments cannot be extrapolated automatically to whole-community assembly. Third, many studies report relative abundance rather than absolute microbial abundance, and most do not jointly measure light environment, plant physiology, root exudates, microbiome composition and agronomic outcomes. The proposed framework is therefore hypothesis-generating rather than a finalized predictive theory.

## 3. Defining the System: Light as Resource, Signal and Rhizosphere Modifier

**Figure 1:**
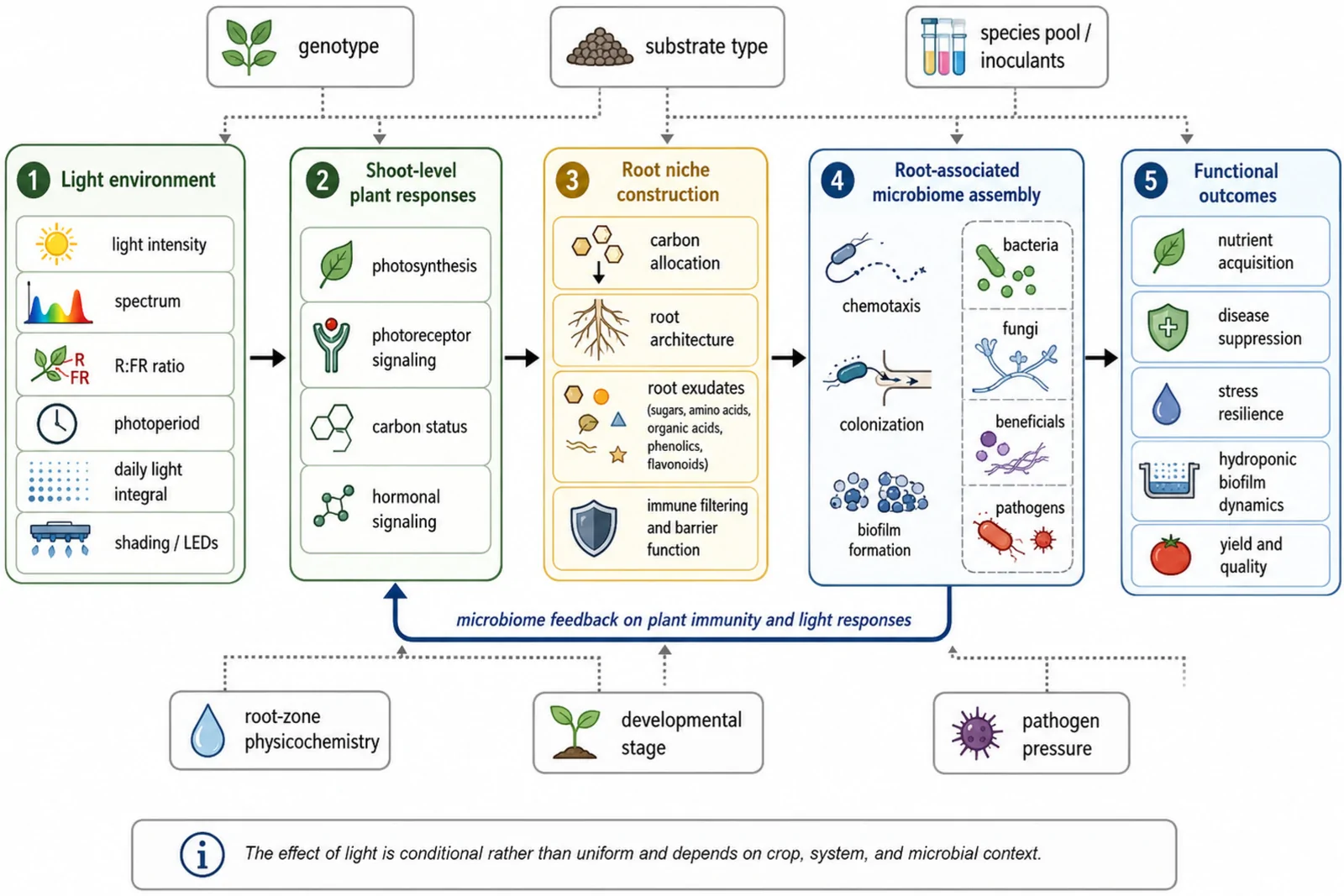
Conceptual model of light-driven root microbiome assembly in protected vegetable crops. The scheme summarizes how light intensity, spectral quality, R:FR ratio, photoperiod, daily light integral, shading, and LEDs influence shoot-level plant responses, root niche construction, root exudation, immune filtering, and root-associated microbiome assembly.

Light has at least three roles in vegetable root-microbiome interactions (Figure 1). First, it is an energy resource. Root exudates may represent a substantial fraction of recently fixed carbon, and this carbon is not ecologically neutral: sugars, organic acids, amino acids, phenolics, flavonoids, coumarins and other metabolites act as microbial substrates, inhibitors, chemoattractants or signals [30,31,32,33,34,35,36,37,38]. Second, light is a developmental signal. Phytochromes, cryptochromes, phototropins and UVR8 modify shoot architecture, root growth, auxin transport, shade avoidance, stress tolerance and immune outputs [39,40,41,42,43,44,45,46,47,48,49,50]. Third, light indirectly modifies the root-zone environment. These effects can all alter microbial niche conditions without requiring microbes to perceive light directly.

A molecular interpretation of this system requires separation between upstream light perception and downstream microbial selection. The HY5–NRT2.1 module provides a clear example of how light information can be converted into root nutrient acquisition, while phosphate-stress and immunity networks show that nutrient signaling can directly alter root microbiota assembly [24,25]. Therefore, light effects on the root microbiome should not be treated as indirect plant-vigor effects only, but as molecularly mediated changes in the biochemical and immunological niche offered by roots.

The root microbiome itself is not a single compartment. Rhizosphere communities are strongly influenced by soil or substrate origin, root exudates and local physicochemical gradients. Rhizoplane communities experience more direct attachment filters, biofilm formation and plant cell-wall proximity. Endosphere communities are subject to immune filtering and tissue-specific nutrient availability. Studies in Arabidopsis, rice and tomato consistently show that microbial diversity and composition differ across bulk soil, rhizosphere and root endosphere [51,52,53,54,55,56,57,58,59]. Therefore, a light treatment that alters rhizosphere substrate supply may not produce the same effect in the root endosphere if immune filtering remains unchanged. A study of a single compartment cannot establish that a light-induced taxon colonized roots, suppressed pathogens or improved nutrient uptake. Stronger inference requires integration of community profiling with exudate chemistry, absolute abundance, culturable isolates, synthetic-community reconstruction and plant-performance assays [60,61,62,63,64,65,66,67,68]. The most persuasive studies in this field combine at least two forms of evidence: community shifts plus metabolite data, community shifts plus pathogen challenge, or colonization assays plus exogenous metabolite complementation [2,4,69].

Protected cultivation of vegetables often replaces complex field soil with coconut coir, rockwool, peat, composted substrates, nutrient-film hydroponics, deep-water culture or sterilized media inoculated by irrigation water and nursery transplants. These systems differ strongly in microbial species pools. Thus, light may regulate microbiome assembly differently in mineral soil than in soilless substrates. In soil, light-induced exudates recruit from a diverse resident reservoir; in hydroponics, the same exudates may enrich a smaller community dominated by water- and surface-associated bacteria. Tomato studies comparing soil and soilless cultivation show that the production system strongly structures bacterial and fungal communities [70]. Lettuce hydroponic studies similarly show that root-zone solution, root endosphere, seed sources and substrate sources contribute differently to community assembly [71,72,73,74].

## 4. Evidence Linking Light with Root-Associated Microbiomes: Direct Vegetable Studies and Mechanistic Analogues

In protected tomato, reduced light intensity altered bacterial and fungal composition in 16S rRNA gene and ITS datasets, including shifts in *Sphingomonas*, *Pseudomonas*, *Mortierella* and a *Gibberella* signal [1]. The experiment did not resolve whether light acted through photosynthesis, temperature, moisture, pathogen pressure or direct microbial exposure, so the taxonomic shifts should not be equated with demonstrated functional improvement. An earlier tomato study crossed light intensity, host genotype and AMF inoculation and used DGGE to profile the rhizosphere bacterial community [6]. Low irradiance reduced AMF colonization in wild-type tomato, and genotype-associated bacterial differences were more pronounced under low than high light.

In tomato, high R:FR promoted root colonization and plant-growth-promoting activity of *Serratia plymuthica* A21-4 through exudate-stimulated chemotaxis and biofilm formation; metabolite complementation supported the proposed causal chain [2]. In tomato and pepper transplants, PGPR-mediated growth promotion by *Bacillus subtilis* GB03 and *Bacillus amyloliquefaciens* IN937a occurred under a 12 h but not an 8 h photoperiod, whereas induced resistance was less dependent on photoperiod [7]. A tomato seedling study further combined an optimized red/blue LED regime with PGPR and AMF and reported treatment-dependent improvements in seedling performance and later yield [13].

The fungal evidence is older and more extensive than the community-sequencing literature. Classical onion experiments showed that irradiance and daylength changed AMF infection, arbuscule development, sporulation, phosphorus uptake and plant growth, with responses depending on light level and phosphorus supply [15,16,17,18]. Modern tomato studies identify a specific spectral mechanism. High R:FR increased AMF colonization and the abundance of the hyphal-branching stimulant 5-deoxystrigol in root exudates [8]. Genetic, grafting and red-light experiments subsequently demonstrated a shoot phyB–mobile HY5–root strigolactone cascade that promoted AMF development and phosphate uptake [12]. Conversely, continuous low R:FR reduced colonization by *Rhizophagus irregularis* and changed AMF effects on tomato growth and defense [75]. In lettuce, five light intensities differentially affected colonization and fungal vesicle abundance and conditioned AMF effects on nutrition and resistance to *Botrytis cinerea* [9]. More recently, tomato root colonization by *Trichoderma harzianum* differed among white, red, blue and red–blue spectra, with the highest colonization under blue light, while plant growth and carbon-metabolism responses depended on the spectrum–inoculation combination [14]. Collectively, these studies demonstrate direct light dependence of defined fungal interactions, not a uniform improvement of the fungal microbiome.

Evidence for non-mycorrhizal beneficial fungi remains much narrower than for AMF. The tomato *T. harzianum* study is currently the clearest direct example: spectral quality changed root colonization together with host carbon-metabolism and rhizosphere-enzyme responses [14]. By contrast, the shading-associated increase in *Mortierella* [1] is a community-level association and cannot establish beneficial function. Accordingly, genus-level ITS shifts should be distinguished from verified colonization or biocontrol outcomes.

In *R. pseudosolanacearum*, deletion of a LOV-domain photoreceptor and changes in illumination affected motility, adhesion, biofilm development and the temporal pattern of tomato root and xylem colonization [10]. This experiment establishes direct microbial light sensing as a parallel mechanism. Its direction is not necessarily beneficial: light-responsive traits can support pathogen localization and virulence. Future vegetable studies should therefore determine whether introduced beneficial strains and resident fungi possess functional photoreceptors and whether light reaches the relevant root-zone microhabitats.

Mechanistic analogues broaden the causal framework but should be labeled explicitly as extrapolations. Rice experiments showed that light–dark versus constant-dark regimes reorganized diel rhizosphere rhythmicity [3]. In Arabidopsis, a bacterial–fungal–oomycete synthetic community supported growth under low photosynthetically active radiation through a MYC2-dependent growth–defense circuit [11], while a separate study implicated FLS2/BAK1, MYC2, PIFs and HY5 in microbiota modulation of shade responses [5]. Diurnal Arabidopsis experiments further linked rhythmic bacterial turnover with rhizosphere carbon metabolism, showed that clock mutants select different communities, and demonstrated clock-dependent reprogramming of both bacterial and fungal rhythms [19,20,21]. Low-light seagrass experiments also associated reduced root exudation with a decline in putatively beneficial root microorganisms [76], supporting carbon-supply sensitivity in a non-terrestrial analogue. In *Panax notoginseng*, light stress induced flavonoids, enriched antagonistic bacteria and generated sterilization-sensitive disease suppression [4]. Shade-duration experiments in *Medicago truncatula* showed that AMF colonization and phosphorus benefits can respond differently to the intensity and duration of carbon limitation [22]. These studies support arrows in the proposed framework, but none substitutes for validation in a protected vegetable system. The evidence studies are summarized in the Table 1.

## 5. Mechanistic Axis I: Photosynthate Allocation and Exudate-Mediated Microbial Recruitment

The most defensible mechanism linking light to root microbiomes is photosynthate allocation. This pathway is supported by decades of rhizosphere carbon research and by modern metabolomics–microbiome studies [30,31,32,33,34,35,36,37,38,77,78,79,80,81,82]. Therefore, the question should be: which compounds are released under a given light regime, and which microbes can use or respond to them? Zhalnina et al. [34] provided a general framework for this question by showing that dynamic root exudate chemistry and microbial substrate preferences can explain patterns of rhizosphere assembly. Guo et al. [2] demonstrated exactly this kind of link for *Serratia plymuthica* A21-4: selected exudate compounds stimulated chemotaxis and biofilm formation, increasing colonization under favorable light quality.

In cucumber, Wen et al. [69] showed that beneficial rhizosphere microbes were enriched through organic acid secretion, linking root exudates to Fusarium wilt resistance. In hydroponic lettuce, different red/blue LED ratios altered root morphology, root activity, total organic carbon secretion and the release of phenolic/autotoxic compounds, including benzoic acid, salicylic acid, gallic acid and tannic acid [83]. Similarly, tomato studies in which root exudation was modified through ethylene-related signaling showed that changes in exudate composition were associated with shifts in bacterial and fungal rhizosphere communities across developmental stages [84].

Rhizosphere signaling studies show that plant-to-microbe, microbe-to-plant and microbe-to-microbe signals operate simultaneously, and PGPR effects on root architecture and nutrient acquisition depend on the compatibility between bacterial traits and the root physiological state [26,27]. For light–root–microbiome research, this means that exudomics should be coupled not only with community profiling but also with microbial behavioral assays, including chemotaxis, biofilm formation, siderophore production and utilization of specific exudate compounds.

Phenolics, flavonoids, coumarins, benzoxazinoids, terpenoids and glucosinolates can recruit or suppress specific microbes [85,86]. In Arabidopsis, coumarin exudation has been linked to microbiome assembly and iron-related plant–microbe interactions [36,37]. In Solanaceae, specialized metabolites such as steroidal glycoalkaloids, phenolics and volatile compounds may influence rhizosphere and endosphere communities [87,88,89]. Tomato roots secrete both primary and specialized metabolites, and tomato exudomics under nutrient deficiency show that root metabolite profiles respond strongly to physiological status [90].

Strigolactones provide the clearest vegetable example of a light-responsive exudate with a defined microbial target. In tomato, high R:FR increased the release of 5-deoxystrigol and AMF colonization [8]. Subsequent mutant, grafting and red-light complementation experiments placed this response in a shoot phyB–mobile HY5–root strigolactone pathway that controlled arbuscule development and mycorrhizal phosphate uptake [12]. This evidence links a photoreceptor, a mobile transcriptional signal, a rhizosphere metabolite and a fungal symbiosis in one causal chain; it should not be generalized automatically to non-mycorrhizal bacteria or to whole-community assembly.

Recent work on photosynthate distribution has shown that recently fixed carbon is not uniformly delivered across the root system and that spatial carbon patterns can influence rhizosphere microbiota [82,91]. Upper roots may experience different oxygen levels, wetting cycles, microbial inocula and exudate concentrations than lower roots. A whole-root homogenized sample can obscure local microbiome responses to light-driven carbon allocation [81,82,91]. Many studies infer exudate effects from plant treatment and microbial composition, but exudate collection is technically difficult. Hydroponic exudates may not represent soil exudates; sterilized systems can distort plant physiology; exudates degrade rapidly; microbes consume compounds during collection; and exudation rates vary with plant size, developmental stage and time of day [79,80,81,82]. Root exudation and microbiome assembly change as plants move from seedling establishment to vegetative expansion and reproductive sink formation [92]. In tomato, rhizocompartment studies under real greenhouse environments show that roots, rhizosphere and bulk soil are distinct habitats even before light is considered [59]. Light treatments imposed at seedling stage may therefore have different microbiome consequences from those imposed at flowering or fruit load. More experiments that combine light treatments, exudomics, microbial chemotaxis or growth assays, synthetic-community validation and plant performance outcomes are necessary to support reliable conclusions [2,4,34,69].

## 6. Molecular Framework Linking Light Perception to Root Microbiome Assembly

**Figure 2:**
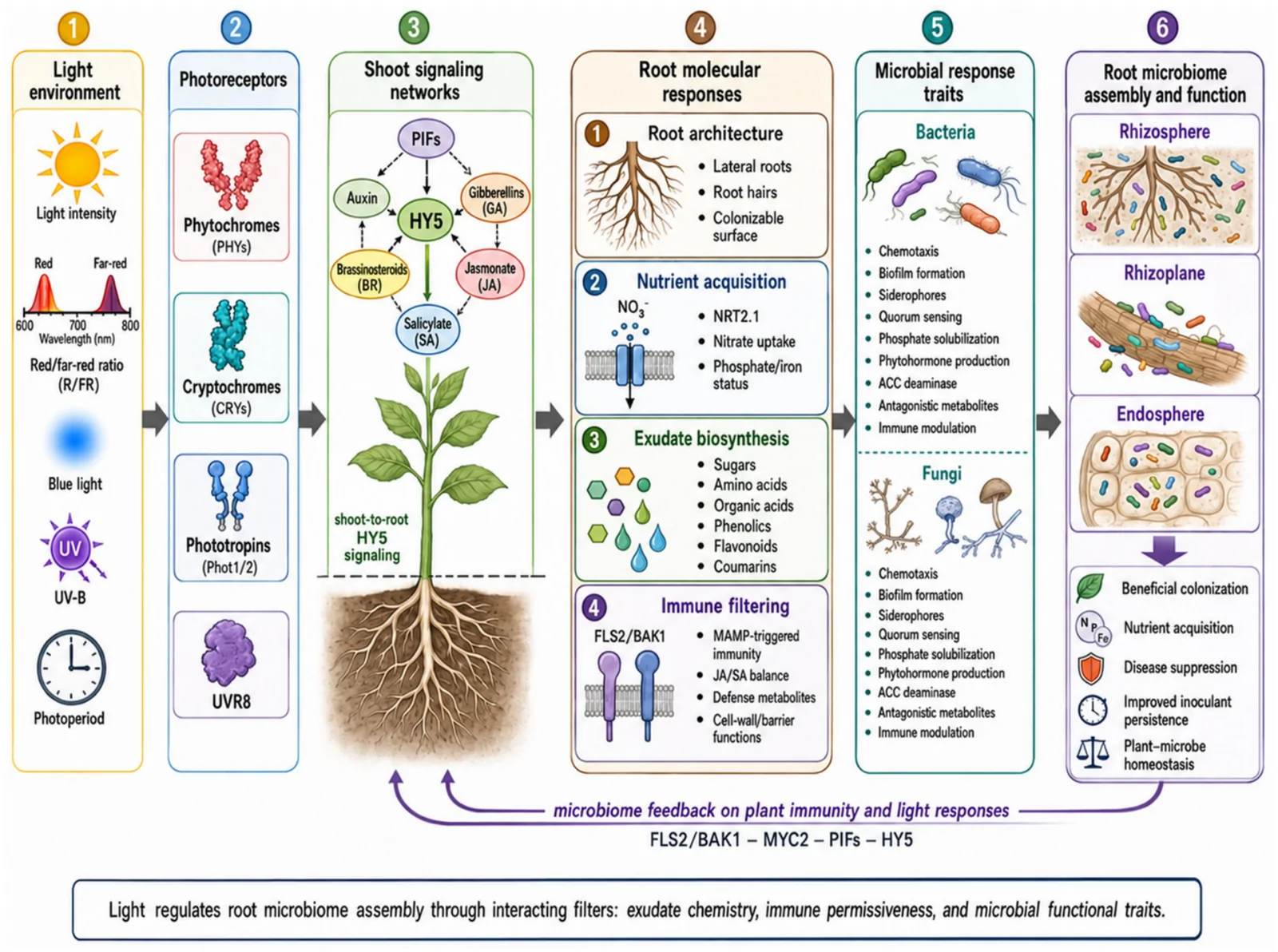
Molecular framework linking light perception, root molecular responses and microbiome assembly in protected vegetable crops. The main sequence represents plant-mediated signaling from photoreceptors through shoot-to-root networks, root architecture, nutrient acquisition, exudate biosynthesis and immune filtering to microbial colonization across the rhizosphere, rhizoplane and endosphere. Direct microbial photoreception constitutes a parallel pathway and is discussed separately. Evidence is strongest for selected links, including R:FR–exudate–*Serratia* colonization and phyB–HY5–strigolactone–AMF signaling; other arrows remain model-system-supported hypotheses requiring vegetable validation.

The most complete vegetable validation is the tomato phyB–HY5–strigolactone pathway: shoot phyB promoted HY5 accumulation and movement to roots, where HY5 activated strigolactone-biosynthesis genes and increased AMF development and phosphate uptake [12]. This pathway substantiates the photoreceptor-to-root-metabolite arrow in Figure 2. By contrast, the extent to which cryptochrome, phototropin or UVR8 signaling alters vegetable root microbiomes remains largely untested.

Shoot-perceived far-red light alters lateral-root development through HY5, while photosynthetic sucrose acts as a long-distance signal controlling early root growth [48,49]. However, the architecture-to-microbiome arrow in Figure 2 remains mainly inferential: no vegetable study has yet isolated root architecture as the mediator between a controlled light treatment and community assembly. A decisive test would combine factorial R:FR or blue-light treatments with three-dimensional root phenotyping, spatially resolved bacterial and fungal profiling, and absolute microbial quantification while holding root-zone temperature and moisture constant.

Mobile HY5 coordinates shoot carbon status with root nitrate uptake through NRT2.1 [24], whereas phosphate-stress and immune pathways jointly shape root microbiota assembly [25]. In tomato, the phyB–HY5 pathway increased strigolactone biosynthesis, AMF development and phosphate uptake, providing direct vegetable evidence for the light-to-nutrient-to-symbiosis connection [12]. Root-secreted coumarins provide a complementary model in which nutrient status and microbial selection interact to improve iron nutrition [38]. Validation therefore requires simultaneous measurements of tissue N, P and Fe, transporter expression, exudate flux, microbial functional genes and isotope-based nutrient transfer in sterile, live and reconstituted communities.

Tomato provides direct evidence for amino-acid- and sugar-mediated recruitment of *Serratia* [2] and for strigolactone-mediated AMF signaling [8,12], whereas *Panax* provides a mechanistic analogue for flavonoid-mediated recruitment of antagonists [4]. Because the same compound classes may stimulate mutualists, commensals or pathogens, exudate shifts require metabolite complementation and strain-level response assays before they can be interpreted as beneficial.

Attraction to the root does not guarantee persistence. Pattern-recognition receptors, JA/SA balance, cell-wall-associated barriers and nutrient-stress pathways filter organisms across the rhizosphere, rhizoplane and endosphere [25,28,29,93]. *Arabidopsis* evidence links shade-responsive signaling with FLS2/BAK1, MYC2, PIFs and HY5 [5,11], but direct validation of these immune gates in vegetable crops is lacking. The corresponding arrow in Figure 2 should therefore be considered model-supported rather than crop-validated. Its validation requires light × microbiome experiments using receptor or hormone perturbations, compartment-resolved absolute abundance and pathogen-challenge endpoints.

Microbial colonization depends on traits such as chemotaxis, motility, adhesion, biofilm formation, nutrient acquisition, antagonistic metabolism and immune modulation [2,26,27]. The *Serratia* study linked several of these traits to host-derived metabolites [2]. However, direct microbial light sensing creates an additional route outside the main plant-mediated chain. A LOV-domain photoreceptor in *R. pseudosolanacearum* regulated motility, adhesion, biofilm development and tomato colonization [10]. Fungal photobiology may likewise influence spores or hyphae near illuminated substrate surfaces, but direct tests in protected vegetable root zones are scarce. Figure 2 should therefore be read as a dominant, not exclusive, causal architecture.

AMF are obligate biotrophs whose colonization and nutrient exchange depend on host carbon supply, rhizosphere signaling and phosphorus status. Historical onion studies established that irradiance and daylength can alter infection, arbuscular activity, sporulation and the plant growth response [15,16,17,18]. In tomato, community-level interactions among irradiance, genotype, AMF and bacteria were demonstrated by Marschner and Timonen [6], while Nagata et al. [8] and Ge et al. [12] resolved R:FR and phyB–HY5–strigolactone mechanisms. Lettuce experiments showed that colonization and AMF-mediated resistance do not track nutrition identically across light intensities [9], and shade-duration experiments in *Medicago* similarly showed partial decoupling of colonization from phosphorus benefit [22]. These results caution against using colonization percentage alone as a proxy for function.

AMF are obligate biotrophs whose light response is closely linked to host carbon supply and strigolactone signaling [8,12,15,16,17,18], whereas the available *Trichoderma* evidence concerns a root-colonizing endophytic/biocontrol interaction assessed through colonization, plant carbon metabolism and rhizosphere enzymes [14]. These contrasting trophic strategies mean that one light regime may enhance mycorrhizal nutrient exchange without producing the same response in non-mycorrhizal endophytes, mycoparasites or pathogenic fungi.

Nevertheless, short-term increases in colonization, root growth or rhizosphere enzyme activity do not establish durable disease suppression. Future studies should combine fungal microscopy, absolute quantification, isolate genomics, pathogen challenge and yield measurements, while separating direct fungal photoreception from plant-mediated carbon and immune effects.

The feedback arrow from microbes to plant light responses is supported most strongly in Arabidopsis. Under suboptimal light, a multikingdom synthetic community redirected a MYC2-dependent growth–defense balance [11], and rhizosphere microbes were later shown to modulate shade-avoidance signaling involving FLS2/BAK1, MYC2, PIFs and HY5 [5]. Host-clock studies further indicate that plant rhythmicity and microbiome rhythmicity are bidirectionally linked [19,20,21]. These experiments justify the feedback arrow conceptually, but the responsible taxa, metabolites and signaling modules have not been validated in tomato, cucumber or lettuce. The arrow should therefore be considered a high-priority vegetable hypothesis rather than an established production mechanism.

## 7. Crop-Specific Evidence and Its Interpretation

Tomato is currently the strongest vegetable model because it spans several evidence types. Community-level studies show light-dependent rhizosphere bacterial and fungal shifts under shading and interactions among irradiance, genotype, AMF and bacterial profiles [1,6]. Interpretation must also account for the species pool because tomato rhizocompartments, cultivar, nursery history and soil versus soilless cultivation independently structure microbial assembly [58,59,70,94,95].

Wen et al. [69] showed that cucumber cultivars differing in Fusarium wilt resistance recruited beneficial rhizosphere microbes through organic acid secretion. Other cucumber studies link rhizosphere microbial communities to Fusarium wilt resistance and susceptibility [96]. A high-value future experiment would cross R/FR or light intensity with resistant and susceptible cucumber genotypes, quantify exuded organic acids, profile microbial communities, isolate candidate antagonists and test Fusarium wilt outcomes.

Direct evidence is available for AMF: five light intensities altered root colonization, fungal vesicle abundance and the balance among nutritional and defense outcomes [9]. In parallel, hydroponic studies show that genotype, salinity, seed transmission, substrate, nutrient solution, pathogen pressure and beneficial bacterial inoculation shape root and endosphere communities [71,72,73,74]. These two evidence streams have not yet been integrated. A decisive experiment would manipulate light while measuring exudates, absolute bacterial and fungal abundance, AMF function, solution biofilms and a root-pathogen endpoint in the same hydroponic system.

Pepper evidence remains narrower. The tomato–pepper transplant study showed that PGPR-mediated growth promotion depended on photoperiod [7], providing direct targeted evidence that lighting can condition bacterial inoculant performance. Separate pepper studies describe cultivar-associated rhizosphere communities and the molecular interaction of *Paenibacillus polymyxa* with the host [87,88], but do not manipulate light. Eggplant appears mainly in cross-Solanaceae disease-suppression work, including evidence that rhizosphere metabolites can protect Solanaceae crops from bacterial wilt by reshaping the soil microbiome [89]. Strawberry root and rhizosphere microbiome studies have linked microbial communities to disease resistance, cultivar, soil health and production practice [97,98]. These crops therefore offer relevant systems for validation, not yet a basis for crop-specific light prescriptions.

## 8. Disease Suppression: From Taxonomic Shifts to Functional Protection

The strongest applied argument for studying light-regulated root microbiomes in vegetables is disease suppression. Chemical control is increasingly limited by regulation, resistance and environmental concerns, while single-strain biological control often fails because introduced microbes do not persist or express the desired function in complex root zones [60,61,62,63,64,65,66,67,68,99,100,101,102,103,104,105].

Stronger inference requires evidence for microbial traits such as antibiosis, lytic enzymes, siderophore-mediated competition, volatile production, induced systemic resistance, quorum-sensing interference, pathogen-growth inhibition, or modulation of host immune markers. Rhizosphere signaling and plant-immunity studies show that beneficial microbes may act either by directly antagonizing pathogens or by recalibrating host immune responses [26,27,28,29]. Therefore, future light-conditioning experiments should include pathogen-load quantification, defense-gene markers, metabolite profiling and isolate- or SynCom-based restoration tests, not only relative-abundance microbiome data.

Disease-suppressive claims require at least one of four forms of evidence: reduced disease in non-sterile conditioned substrate; loss of suppression after sterilization or microbiome disruption; restoration of suppression by microbial consortia or isolates; or direct antagonism/colonization mechanisms linked to plant outcomes. Plants under biotic or abiotic stress can alter exudation and recruit beneficial microbes that improve defense or stress tolerance [93,106,107]. Fang et al. [4] approached this standard by combining light treatments, microbial community profiling, root metabolomics, pathogen analysis, sterilization and isolate testing. Tomato shading work [1] is suggestive but should be followed by pathogen-challenge assays before making disease-control claims. In vegetables, the challenge is to separate useful elicitation from yield penalty.

In lettuce, light intensity conditioned AMF colonization and AMF-associated resistance to the foliar pathogen *Botrytis cinerea*, but nutritional and resistance responses were partly uncoupled [9]. In tomato, light and a bacterial LOV photoreceptor directly regulated colonization and virulence traits of *R. pseudosolanacearum* [10].

Organic acids can recruit antagonists, solubilize nutrients or favor pathogenic fungi depending on concentration and community context. Flavonoids can suppress pathogens, stimulate beneficial bacteria or change microbial gene expression in unexpected ways [4,86,87,88,89]. Therefore, light-induced exudation should be evaluated under pathogen pressure, not only in healthy plants. The same light recipe may look beneficial in a sterile hydroponic assay and harmful in pathogen-infested substrate if it enriches opportunistic microbes.

A key unresolved issue is stability. A disease-suppressive microbiome induced by light in seedlings may not persist through flowering and fruiting. Vegetable crops undergo major changes in sink strength, root turnover and exudation during development. Tomato rhizosphere communities shift over time and can be influenced by transplanting, grafting, microbial amendments and fruiting stage [94,95,108]. Thus, light-induced microbiome changes need longitudinal evaluation. Sampling only at one vegetative stage risks overestimating stability and underestimating late-season disease vulnerability.

## 9. Nutrient Acquisition, Substrate Cycling and Hydroponic Root-Zone Function

Root-associated microbes influence nitrogen transformations, phosphorus solubilization, iron mobilization, sulfur cycling, hormone production and stress tolerance [99,100,101,102,103,104]. In tomato, shading enriched Mortierella and bacterial genera associated with nutrient transformation [1]. Therefore, light, P fertilization and microbiome assembly are likely to interact. Tomato experiments showed that R:FR and the phyB–HY5–strigolactone pathway regulate arbuscular development and mycorrhizal phosphate uptake [8,12]. Accordingly, future studies should quantify both colonization and functional nutrient fluxes; a larger fungal presence does not necessarily imply a larger phosphorus benefit.

Iron acquisition provides another mechanistic example. Coumarins and other phenolic compounds can influence iron mobilization and shape the root microbiome [36,37,38]. Tomato and leafy vegetables frequently encounter iron availability problems in high-pH substrates or recirculating systems. Because light can change photosynthetic demand and secondary metabolism, it may alter the production of iron-mobilizing compounds and the microbes that consume or modify them. The outcome could be beneficial if microbes enhance iron availability, but detrimental if microbes degrade plant-secreted chelators and compete for iron [38]. Thus, light-induced recruitment should be assessed functionally rather than assumed to be positive.

Hydroponic and soilless vegetable systems deserve special attention because their root-zone microbiomes are more exposed to management. In nutrient-film technique, deep-water culture and recirculating drip systems, microbes colonize roots, channels, tanks, emitters and biofilms. Lettuce studies show that hydroponic root and solution microbiomes can be shaped by seed, substrate and inoculant sources [71,72,73,74]. Tomato soil versus soilless comparisons show that substrate changes fungal and bacterial assembly during the cultivation chain [70]. Light recipes in these systems may influence root exudation into a shared nutrient solution, potentially altering biofilm development and microbial dispersal.

Rice and Arabidopsis studies show that light cycles, host-clock function and time of sampling can restructure bacterial and fungal rhythmicity [3,19,20,21]. Hydroponic lighting experiments should therefore standardize sampling relative to lights-on and include repeated measurements across the photoperiod rather than treating the root-zone microbiome as temporally static. Environmental modulation of rhizodeposition also means that nutrient and water context cannot be treated as background noise. Soil studies have shown that microbial responses to rhizodeposition depend on light intensity and soil nutrient availability [109], while more recent work has explicitly asked whether root exudate shifts induced by fluctuating light and water stress recruit particular rhizosphere microbes [110]. Trait-based exudate studies further show that exudation is linked to functional plant traits rather than being a fixed species property [77]. These findings support a more quantitative view of protected vegetable systems: light recipes, fertigation and water status jointly determine the biochemical niche that roots provide to microbes (Table 2).

## 10. Knowledge Gaps and Priority Research Questions

Community-level evidence remains restricted mainly to tomato shading and irradiance–genotype–AMF experiments [1,6]. In contrast, targeted studies cover PGPR, AMF, *Trichoderma* and a bacterial pathogen across tomato, pepper and lettuce [2,7,8,9,10,11,12,13,14,75]. These studies resolve particular mechanisms but cannot establish how a complete bacterial–fungal community responds or whether the effect persists through a production cycle. The priority is therefore to connect targeted mechanisms with multikingdom community profiling and agronomic outcomes in the same experiment.

What remains scarce is simultaneous analysis of AMF, beneficial non-mycorrhizal fungi, pathogenic fungi, oomycetes and bacteria under the same light treatment. Future studies should combine ITS profiling with AMF microscopy, fungal isolation, pathogen-specific qPCR, absolute fungal biomass and re-inoculation or SynCom tests. This is necessary because relative ITS enrichment of *Mortierella*, *Trichoderma* or a pathogen-associated genus cannot by itself establish function.

The tomato irradiance experiment indicates that AMF status can coincide with changes in bacterial community structure [6], while the low-light Arabidopsis SynCom demonstrates that bacteria, fungi and oomycetes can jointly determine plant responses [11]. Vegetable studies should therefore test bacteria, AMF and non-mycorrhizal fungi together rather than evaluating each kingdom in isolation.

The third gap is strain-level function. Metagenomics can guide this work, but culture collections remain essential for causal tests. A related molecular gap is the lack of functional-gene and expression-level evidence. Amplicon sequencing can identify community shifts, but it cannot determine whether light-enriched microbes express genes for chemotaxis, biofilm formation, nutrient solubilization, siderophore biosynthesis, phytohormone production, immune modulation or antagonism. Future experiments should therefore combine 16S/ITS profiling with shotgun metagenomics, metatranscriptomics, qPCR of strain-specific and functional genes, exometabolomics and genome sequencing of cultured isolates. Synthetic communities are particularly useful because they allow candidate strains to be reassembled under defined light regimes and tested for colonization, immune modulation, nutrient acquisition and pathogen suppression [28,29,62,63]. This would move the field from descriptive light-associated microbiome patterns toward causal molecular mechanisms.

The fourth gap is temporal dynamics. Vegetable crops differ in production duration and developmental trajectory, and light effects on exudation and microbial recruitment are likely to change between seedling establishment, vegetative growth, flowering and fruiting. The circadian dimension is also experimentally supported: rice and Arabidopsis studies show diel community turnover, host-clock effects and rhythmicity of both bacterial and fungal taxa [3,19,20,21]. Vegetable experiments therefore require both developmental time series and standardized within-day sampling.

Many mechanistic studies use sterilized agar, hydroponics or small pots. These systems are useful but not sufficient. Protected vegetables are grown in grafted combinations, reused substrates, fertigation systems, high humidity and variable canopy structures. Validation in commercial or semi-commercial greenhouse systems will be required before recommendations are credible. Conversely, greenhouse studies without mechanistic measurements will remain descriptive. The field needs both controlled and realistic experiments linked by common hypotheses.

The sixth gap is integration with food safety. Lettuce, tomato and other fresh vegetables can harbor human-associated bacteria in root-zone or leaf-associated habitats. Light management may influence plant-associated microbial communities beyond agronomic microbes [111,112]. This does not mean light creates food-safety risk, but it does mean that microbiome steering in leafy vegetables should consider undesirable taxa, not only beneficial PGPR. Root-zone solution management, biofilm control and microbial inoculant quality are relevant endpoints.

**Figure 3:**
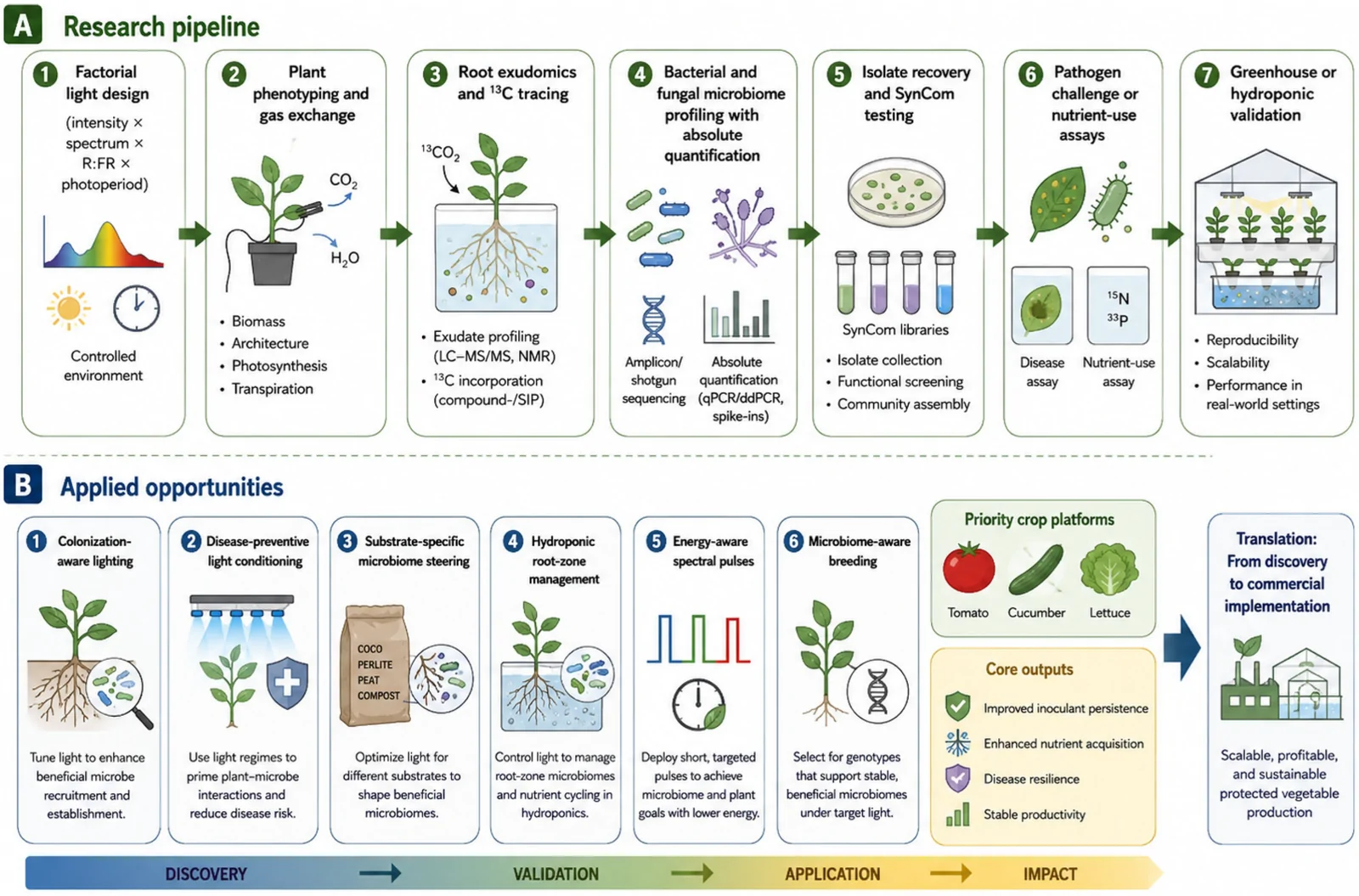
Research and application roadmap for microbiome-aware lighting in protected vegetable production. The roadmap outlines a staged research pipeline from factorial light treatments and plant phenotyping to exudomics, microbiome profiling, isolate recovery, synthetic-community testing, pathogen or nutrient-use assays, and greenhouse or hydroponic validation. It also summarizes applied opportunities, including colonization-aware lighting, disease-preventive light conditioning, substrate-specific microbiome steering, hydroponic root-zone management, energy-aware spectral pulses, and microbiome-aware breeding.

The seventh gap is quantitative modeling. The field needs models that connect daily light integral, R/FR ratio, photosynthesis, root exudation, microbial growth and plant performance. Such models could predict when a light change is large enough to affect rhizosphere carrying capacity or when a spectral cue is likely to alter exudate composition. Current microbiome studies are often statistical rather than predictive. Combining plant growth models with microbial trait models and metabolite flux data would help turn empirical observations into management principles. The practical roadmap for future research is presented in Figure 3.

## 11. Conclusions

The vegetable root microbiome is connected to the canopy light environment, but the strength of evidence differs by endpoint. A broader targeted literature shows that light intensity, photoperiod and spectral quality can condition PGPR establishment, AMF symbiosis, beneficial fungal colonization and pathogen behavior [2,7,8,9,10,11,12,13,14,75]. These results demonstrate conditional light sensitivity of particular plant–microbe interactions; they do not establish a universal spectrum that improves the whole root microbiome.

Within the fungal component, mechanistic support is strongest for AMF, whereas evidence for non-mycorrhizal beneficial fungi is currently limited to a small number of targeted experiments and should not be generalized to fungal community function [8,9,12,14,15,16,17,18,75].

High R:FR altered tomato exudates and *Serratia* colonization [2]; shoot phyB and mobile HY5 regulated root strigolactones, AMF development and phosphate uptake [8,12]; and a bacterial LOV photoreceptor directly regulated *Ralstonia* colonization traits [10]. Model systems further support microbiota feedback under low light and host-clock control of microbial rhythmicity [3,5,11,19,20,21]. Together, these findings extend the framework beyond carbon supply alone to include metabolite-specific signaling, immune filtering, microbial response traits, direct microbial photoreception and temporal regulation.

For protected production, microbiome-aware lighting remains a testable strategy rather than a validated prescription. Lighting, substrate and inoculant design should be evaluated together because the same light cue can favor a beneficial symbiont, have no durable effect, or modify pathogen behavior depending on the microbial partner and root-zone context. The decisive next step is factorial validation that integrates plant physiology, exudomics, absolute multikingdom quantification, isolate or SynCom reconstruction, nutrient flux, pathogen challenge and yield.

## Figures and Tables

**Table 1:** Direct vegetable evidence and selected mechanistic analogues linking light with root-associated microbial communities, colonization and function.

Crop/Model	Light Factor	Main Experimental Evidence	Microbial Endpoint or Mechanism	Evidence Class/Interpretation	Refs.
Tomato	Reduced light intensity/shading	16S rRNA and ITS profiling detected compositional shifts in the rhizosphere.	*Sphingomonas*, *Pseudomonas*, *Mortierella* and *Gibberella*-related signals.	Direct community evidence; function was not demonstrated.	[1]
Tomato	Low versus high irradiance	Light, tomato genotype and AMF inoculation interacted to alter DGGE bacterial profiles and AMF colonization.	Rhizosphere bacterial community and AMF colonization.	Direct community evidence with lower taxonomic resolution.	[6]
Tomato	High versus low R:FR	High R:FR enhanced *Serratia plymuthica* A21-4 chemotaxis, biofilm formation and root colonization through exudate changes.	Defined PGPR colonization.	Direct targeted causal evidence; strain-specific.	[2]
Tomato/pepper	8 h versus 12 h photoperiod	PGPR-mediated growth promotion occurred under the longer photoperiod, whereas induced resistance was less light-dependent.	*Bacillus subtilis* GB03 and *B. amyloliquefaciens* IN937a.	Direct targeted evidence for photoperiod-dependent inoculant performance.	[7]
Tomato	R:FR and red-light signaling	High R:FR and phyB–HY5 signaling increased strigolactone production, AMF colonization and phosphate uptake.	AMF hyphal branching, arbuscules and mycorrhizal P pathway.	Direct molecular evidence from exudates, mutants, grafting and complementation.	[8,12]
Tomato	Continuous low R:FR	Low R:FR reduced *Rhizophagus irregularis* colonization and changed AMF effects on shade avoidance and defense.	AMF colonization and host defense responses.	Direct targeted evidence showing context-dependent, not uniformly beneficial, effects.	[75]
Lettuce	Five light intensities	Light intensity altered AMF colonization, vesicle abundance, nutrition and AMF-associated resistance to *Botrytis cinerea*.	AMF colonization and induced resistance.	Direct targeted vegetable evidence.	[9]
Tomato	Optimized red/blue LED regime	LED treatment interacted with PGPR and AMF inoculation to improve seedling performance and later yield.	PGPR and AMF inoculants.	Supportive applied evidence; multifactorial and not a community study.	[13]
Tomato	White, red, blue and red–blue light	Root colonization by *Trichoderma harzianum* and plant carbon-metabolism responses differed among spectra.	*T. harzianum*, root traits and rhizosphere enzymes.	Direct beneficial-fungus evidence; short-term seedling study.	[14]
Tomato	Light versus darkness; LOV photoreceptor	Light sensing regulated pathogen motility, adhesion, biofilm formation and root/xylem colonization.	*Ralstonia pseudosolanacearum* virulence traits.	Direct microbial photoreception; demonstrates a potentially harmful pathway.	[10]
Onion	Irradiance and daylength	Light altered AMF infection, arbuscules, sporulation, P uptake and growth, with strong nutrient dependence.	Vesicular-arbuscular mycorrhiza.	Historical direct symbiosis evidence.	[15,16,17,18]
Rice/Arabidopsis	Light–dark cycles, low PAR and host-clock perturbation	Light and host circadian function structured microbial rhythmicity; a multikingdom SynCom modified growth–defense allocation under low light.	Rhythmic bacterial/fungal assembly and microbiota feedback.	Mechanistic analogues; vegetable validation required.	[3,11,19,20,21]
*Panax notoginseng*	Suboptimal light intensity	Light-induced flavonoids enriched antagonists and generated sterilization-sensitive root-rot suppression.	Antagonistic bacteria and lower pathogen pressure.	Strong mechanistic analogue for disease suppression.	[4]

**Table 2:** Key findings, evidence status, remaining uncertainties and decisive experiments in light–root–microbiome research.

Mechanistic Link	Key Finding	Evidence Status	Remaining Uncertainty	Decisive Experiment/Measurement
Light intensity/community composition	Tomato rhizosphere communities changed under shading; irradiance interacted with tomato genotype and AMF.	Direct community evidence [1,6].	Drivers and functional consequences remain unresolved.	Factorial light × temperature × moisture designs with absolute bacterial/fungal abundance and functional endpoints.
Light/root architecture/microbial habitat	Far-red–HY5 and photosynthate signals alter lateral-root development and root growth; architecture structures microbial habitats and exudation zones.	Strong plant-mechanistic evidence [48,49,91]; direct mediation of vegetable microbiome assembly has not been demonstrated.	Architecture may covary with exudation, development and root-zone physicochemistry, making its independent contribution unclear.	Factorial light experiments with 3D root phenotyping, spatial microbiome profiling, absolute abundance and mediation analysis under controlled root-zone conditions.
Light/nutrient acquisition/microbiome filtering	HY5 coordinates nitrate uptake, phyB–HY5 regulates strigolactone-dependent AMF and P uptake, and nutrient-stress pathways interact with microbiota and immunity.	Direct tomato evidence for AMF/P [12]; model-system evidence for nitrate, phosphate-stress and iron-related microbiota interactions [24,25,38].	Generality across N, P and Fe regimes, vegetable species and microbial partners remains unresolved; nutrient change may be cause or consequence.	Paired isotope tracing, transporter expression, exudomics and microbial functional profiling in sterile, native and reconstituted communities.
R:FR/bacterial colonization	High R:FR promoted *Serratia* chemotaxis, biofilm formation and root colonization through exudate changes.	Direct strain-level causal evidence [2].	Generality across strains, cultivars and substrates is unknown.	R:FR × strain × substrate tests with exudomics, complementation and absolute colonization.
R:FR/AMF signaling	R:FR and phyB–HY5 signaling regulated strigolactones, AMF development and phosphate uptake in tomato.	Direct molecular evidence [8,12,75].	Responses may differ among AMF species and nutrient regimes.	Multi-AMF experiments with mutants, strigolactone flux, arbuscule activity and 33P or equivalent P-tracing.
Light intensity/fungal function	Light altered AMF colonization and the balance among growth, nutrition and resistance in lettuce and other hosts.	Direct lettuce evidence plus analogues [9,15,16,17,18,22].	Colonization and benefit can be decoupled.	Simultaneous microscopy, fungal biomass, nutrient flux, pathogen challenge and yield measurements.
Photoperiod/inoculant performance	PGPR-mediated growth promotion in tomato and pepper depended on photoperiod.	Direct targeted evidence [7].	Mechanism and persistence in production substrates are unclear.	Photoperiod × inoculant trials with root-zone chemistry, strain-specific qPCR and repeated colonization measurements.
Circadian and diel control	Host light cycles and clock function structured rhythmic bacterial and fungal assembly.	Strong model-system evidence [3,19,20,21].	Vegetable crops and controlled-environment photoperiods remain untested.	Clock-aware time series with paired exudomics and microbiome profiling.
Direct microbial photoreception	A LOV photoreceptor regulated *Ralstonia* motility, biofilm formation and tomato colonization.	Direct pathogen evidence [10].	Prevalence and importance of photoreceptors in beneficial root microbes are unknown.	Photoreceptor mutants, in situ light measurements and colonization assays for beneficial and pathogenic strains.
Low light/microbiome feedback	Multikingdom microbiota modified growth–defense allocation and shade responses in Arabidopsis.	Mechanistic analogue [5,11].	Taxa and signaling modules have not been validated in vegetables.	Vegetable SynCom experiments under low PAR or low R:FR with MYC2, HY5, JA/SA and defense endpoints.
Beneficial non-mycorrhizal fungi/cross-kingdom interactions	*Trichoderma* colonization and host carbon/rhizosphere-enzyme responses varied among spectra, whereas *Mortierella* enrichment under tomato shading remains correlative.	One direct short-term tomato study plus community-level association [1,14].	Generality across endophytes and mycoparasites, long-term persistence, disease suppression and bacterial–fungal interactions remain unresolved.	Longitudinal qPCR or microscopy, fungal isolate/SynCom assays, pathogen challenge and bacterial–fungal co-inoculation under matched spectra.
Production-system context	Soil, substrate, hydroponics, nursery history and species pool strongly condition microbiome assembly.	Strong indirect vegetable evidence [58,59,70,71,72,73,74].	The same light treatment may recruit different organisms in different systems.	Matched soil, substrate and hydroponic validation with identical light treatments and inoculum tracking.

## Data Availability

No new data were created or analyzed in this study. Data sharing is not applicable to this article.
